# Supplementary material for: Improving cell-free glycoprotein synthesis by characterizing and enriching native membrane vesicles
Source: Nat Commun. 2021 Apr 22;12:2363. doi: 10.1038/s41467-021-22329-3 (PMC8062659; doi:10.1038/s41467-021-22329-3)
Supplement: Supplementary file 1 — Supplementary Information [file 41467_2021_22329_MOESM1_ESM.pdf]

Supplementary Information for

Improving cell-free glycoprotein synthesis by characterizing and enriching native membrane vesicles

Jasmine M. Hershewe[a,b,c,1], Katherine F. Warfel [a,b,c,1], Shaelyn M. Iyer[a], Justin A. Peruzzi [a,b,c], Claretta J. Sullivan[d], Eric W. Roth[e], Matthew P. DeLisa[f,g,h], Neha P. Kamat[b,c,i], and Michael C. Jewett [a,b,c,j,k 2]

Affiliations

[a]Department of Chemical and Biological Engineering, Northwestern University, 2145 Sheridan Road, Technological Institute E136, Evanston, IL 60208, USA
[b]Chemistry of Life Processes Institute, Northwestern University, 2170 Campus Drive, Evanston, IL 60208, USA
[c]Center for Synthetic Biology, Northwestern University, 2145 Sheridan Road, Technological Institute E136, Evanston, IL 60208, USA
[d]Air Force Research Laboratory, Materials and Manufacturing Directorate, Wright-Patterson Air Force Base, Dayton, OH 45433, USA
[e]Northwestern University Atomic and Nanoscale Characterization and Experimentation (NU*ANCE*) Center, 2145 Sheridan Road, Tech Institute A/B Wing A173, Evanston, IL 60208, USA
[f]Robert F. Smith School of Chemical and Biomolecular Engineering, Cornell University, Ithaca, New York 14853, USA
[g]Nancy E. and Peter C. Meinig School of Biomedical Engineering, Cornell University, Ithaca, NY 14853, USA
[h]Biomedical and Biological Sciences, College of Veterinary Medicine, Cornell University, Ithaca, New York 14853, USA
[i]Department of Biomedical Engineering, Northwestern University, 2145 Sheridan Road, Technological Institute E310, Evanston, IL 60208, USA
[j]Robert H. Lurie Comprehensive Cancer Center, Northwestern University, 676 North Saint Clair Street, Suite 1200, Chicago, IL 60611, USA
[k]Simpson Querrey Institute, Northwestern University, 303 East Superior Street, Suite 11-131, Chicago, IL 60611, USA

[1]Authors contributed equally
[2]To whom correspondence should be addressed
*Michael C. Jewett
Phone: 1 847-497-5007
Email: m-jewett@northwestern.edu

Legend and contents of supplementary information:

Supplementary Figure 1. Production of superfolder green fluorescent protein (sfGFP) in standard cell-free expression (CFE) reaction conditions.

Supplementary Figure 2. Purification and characterization of membrane vesicles from CFE extracts.

Supplementary Figure 3. Cryo-EM analysis of vesicles in crude extracts (top) and after SEC purification (bottom).

Supplementary Figure 4. CFE productivities of all S30 (white) and S12 (gray) extracts characterized in this study.

Supplementary Figure 5. Additional light scattering characterization of extracts presented in Figure 3 of the main text.

Supplementary Figure 6. Western blot densitometry analysis of membrane protein enrichment in S30 and S12 extracts.

Supplementary Figure 7. Characterization of vesicle and PglB enrichment in CFGpS extracts.

Supplementary Figure 8. Fluorescent probes confirm that glycosylation components are associated with membrane vesicles in CFE extracts.

Supplementary Figure 9. Characterization of *N*-linked glycosylation in S12 and S30 CFGpS extracts.

Supplementary Figure 10. Residue-specific glycosylation of acceptor proteins with permissible sequons for PglB and PglO.

Supplementary Figure 11. LC-MS/MS of trypsin digested glycopeptides.

Supplementary Figure 12. Characterization of *O*-linked glycosylation in CFGpS extracts.

Supplementary Figure 13. Characterization of *N*-linked glycosylation with various acceptor proteins in CFGpS extracts using a ^{14}C-L incorporation assay.

Supplementary Table 1. Information on proteins selected for extract enrichment in this study.

Supplementary Table 2. Acceptor protein coding sequences used in CFGpS reactions.

Supplementary Table 3. Strains and plasmids used in this study.

Supplementary Table 4. Information on primers used to construct plasmids made for this study.

Supplementary Methods

Supplementary References

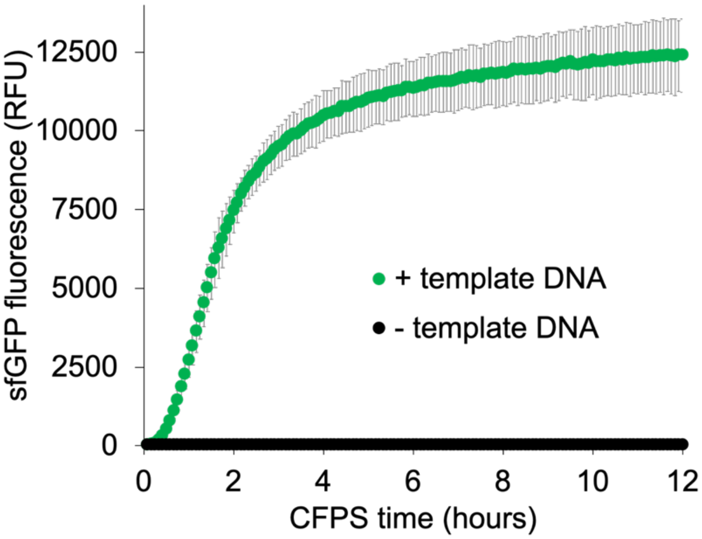

Supplementary Figure 1. Production of superfolder green fluorescent protein (sfGFP) in standard cell-free expression (CFE) reaction conditions. Protein synthesis proceeds when template DNA is present (green), and no fluorescence is observed when template DNA is omitted (black). Data are presented as mean values +/- SD of n=3 biologically independent CFE reactions. Source data for all panels are provided as a Source Data file.

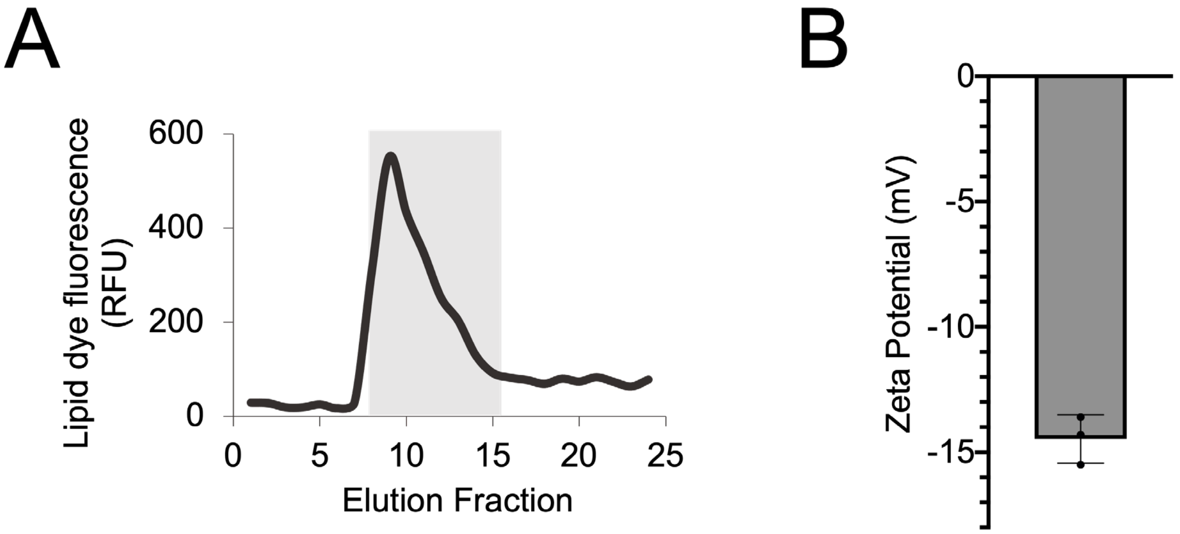

Supplementary Figure 2. Purification and characterization of membrane vesicles from CFE extracts. (A) SEC chromatogram of extracts probed with FM 4-64 lipid dye. The gray segment indicates the characteristic vesicle elution fraction. (B) Zeta potential analysis of purified vesicles in PBS. Data are presented as mean values +/- SD of n=3 Zeta Sizer measurements of purified vesicles. Data are representative of three independent experiments. Source data are provided as a Source Data file.

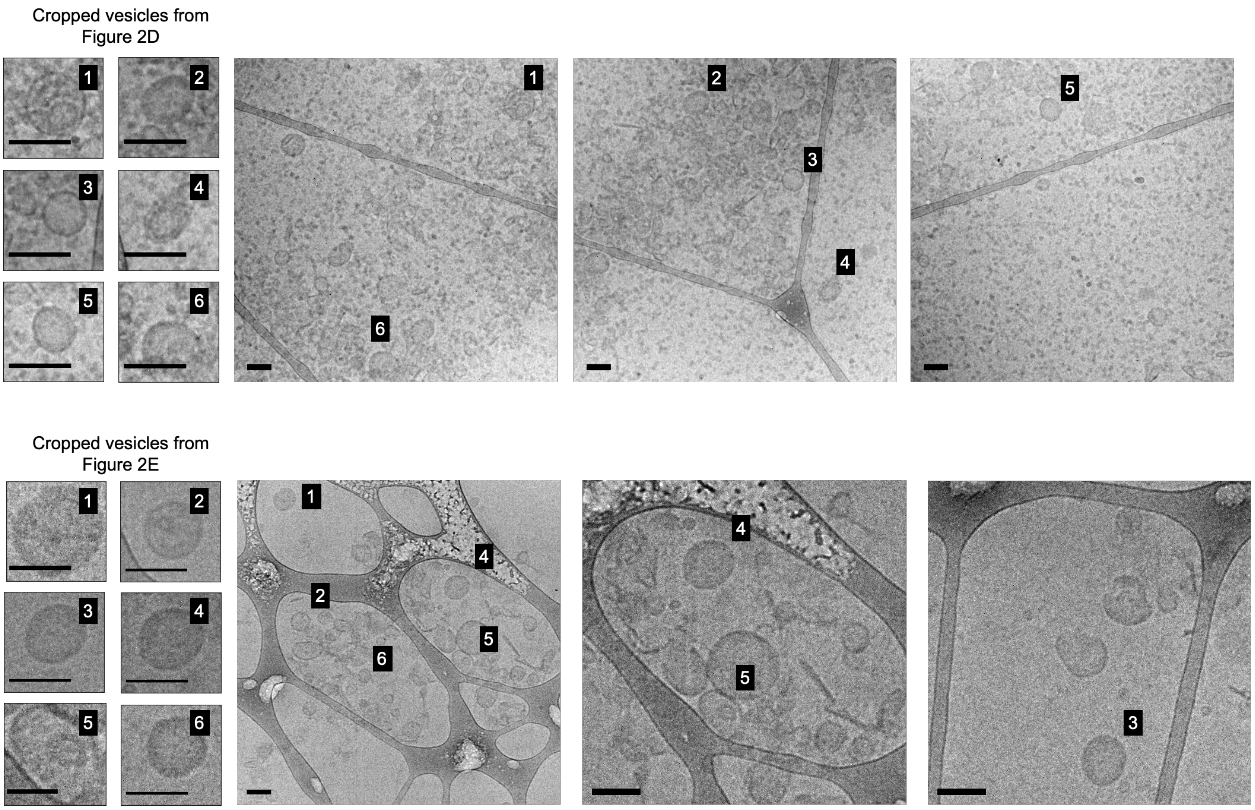

Supplementary Figure 3. Cryo-EM analysis of vesicles in crude extracts (top) and after SEC purification (bottom). The numbering of vesicles in uncropped images corresponds with the vesicle shown in each cropped image in Figure 2 of the main text. All scale bars are 100 nm. Data are representative of four independent experiments. Source data are provided as a Source Data file.

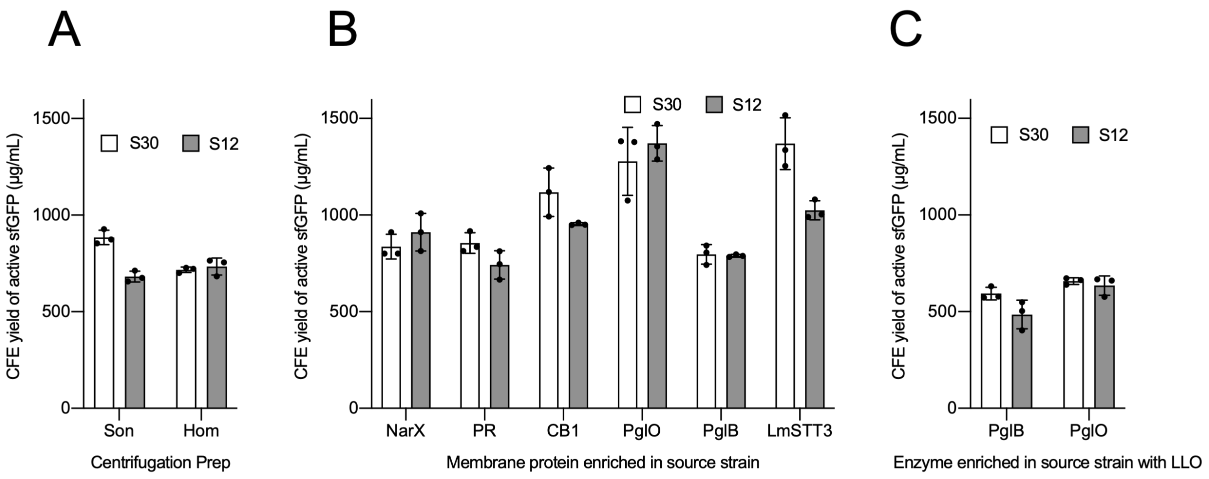

Supplementary Figure 4. CFE productivities of all S30 (white) and S12 (gray) extracts characterized in this study. Reactions were run for 20 hours at 30 °C under standard conditions. (A) Extracts made from 'blank' chassis strains with no overexpressed components. These extracts are characterized in Figures 2 and 3 of the main text. (B) Extracts enriched with membrane proteins, characterized in Figure 4 of the main text. (C) CFGpS extracts with enrichment of *C. jejuni* LLO and the denoted enzyme in the strain. CFGpS extracts are characterized in Figure 5 of the main text. Data in A-C are presented as mean values +/- SD of n=3 biologically independent CFE reactions. Source data for all panels are provided as a Source Data file.

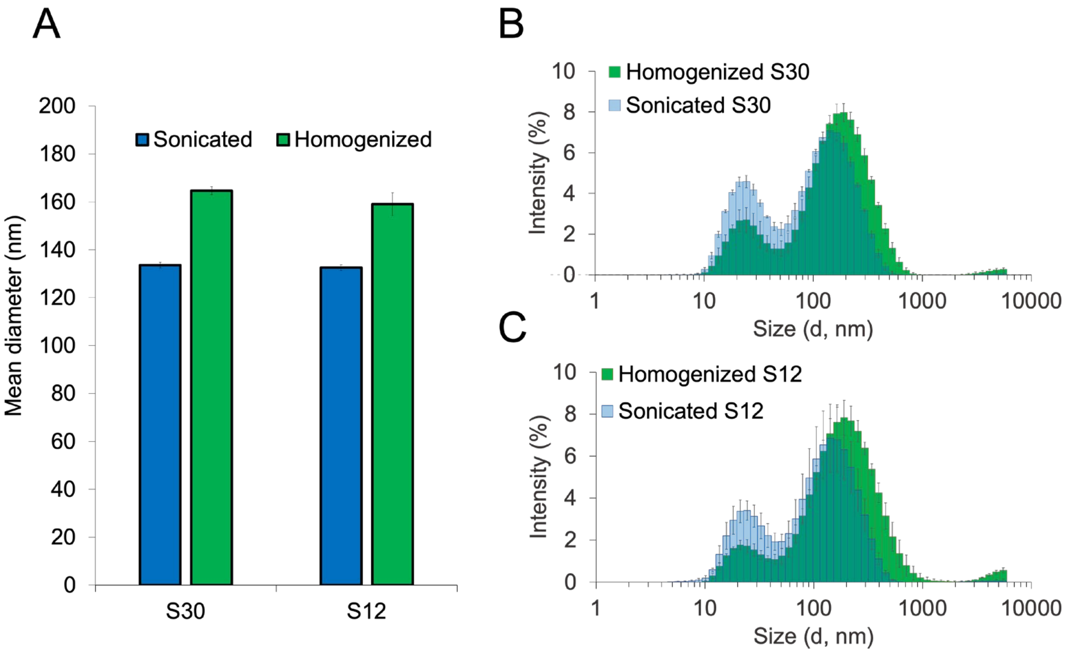

Supplementary Figure 5. Additional light scattering characterization of extracts presented in Figure 3 of the main text. (A) Mean diameters of vesicles in sonicated (blue) and homogenized (green) extracts, determined by NTA. Data are presented as mean value +/- SD of n=15 replicates (3 biologically independent extracts, each examined over 5, 1-minute NTA measurements). DLS analysis of (B) S30 extracts and (C) S12 extracts. Sonicated extract data is in blue and homogenized extract data is in green. Spectra corroborate larger, right-shifted vesicle peaks in homogenized extracts in both cases. Consistent with NTA particle counting, the relative peak heights of ~20 nm peak (ribosomes/small cellular complexes) to vesicle peak indicates that homogenized extracts contain higher concentrations of vesicles than sonicated extracts for each given prep method. DLS data are presented as mean intensity values for a given size +/- SD of n=9 measurements (3 biologically independent extracts, each measured 3 times). Source data for all panels are provided as a Source Data file.

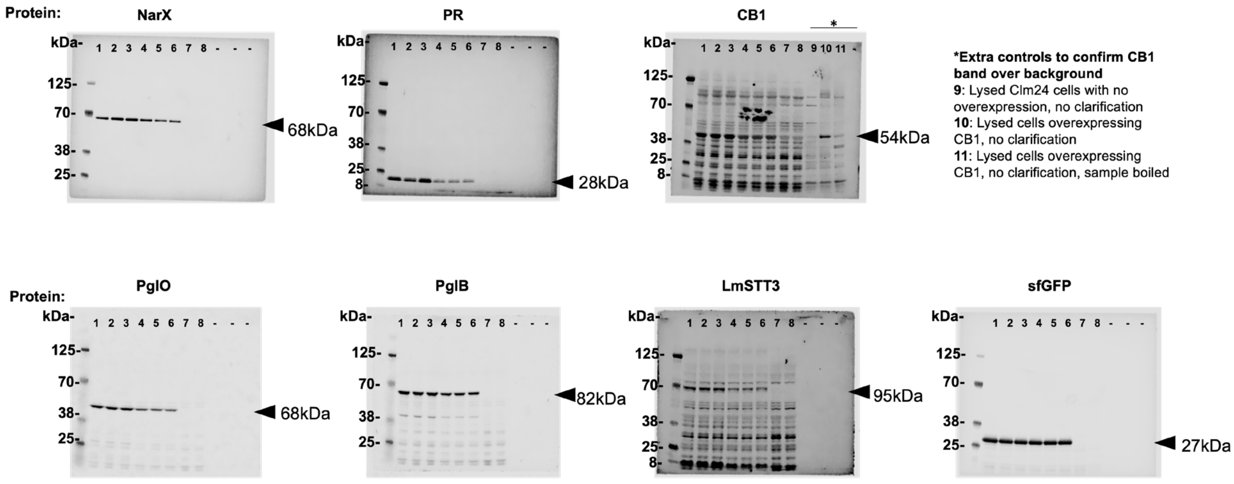

Lane	S12/S30	Extract replicate (#)	Notes
Ladder	-	-	-
1	S12	1	-
2	S12	2	-
3	S12	3	-
4	S30	1	-
5	S30	2	-
6	S30	3	-
7	S12	N/A	No overexpression, strain background control
8	S30	N/A	No overexpression, strain background control

Protein	S12 avg densitometry signal	S12 std dev densitometry signal	S30 avg densitometry signal	S30 std dev densitometry signal	S12/S30 densitometry signal	S12/S30 error densitometry signal	S12/S30 ratio rounded for Figure
NarX	2.76	0.70	1.44	0.37	1.91	0.69	1.9
PR	0.83	0.22	0.21	0.03	3.88	1.16	3.9
CB1	0.25	0.01	0.13	0.01	1.88	0.11	1.9
PglO	8.71	0.36	4.66	0.37	1.87	0.17	1.9
PglB	5.23	0.10	3.47	0.18	1.51	0.08	1.5
LmSTT3	0.16	0.01	0.08	0.01	2.14	0.12	2.1
sfGFP	16.87	0.72	16.90	0.26	1.00	0.05	1.0

Supplementary Figure 6. Western blot densitometry analysis of membrane protein enrichment in S30 and S12 extracts. Uncropped α-FLAG blots against each of the indicated recombinant proteins. The theoretical mass of each recombinant protein is listed next to black arrows, indicating the corresponding band. Molecular weight markers are labeled to the left in kDa. We observe the well-documented effect that membrane proteins run anomalously on SDS-PAGE, running 'light' with respect to the protein ladder standard. A lane key is presented below blots. Note that extra controls (indicated to the right of blot) were needed for the CB1 blot to confirm the presence of the protein. Each Western blot includes n=3 biologically independent S12 extracts and n=3 biologically independent S30 extracts that were used to generate densitometry measurements. Averages and standard deviations of densitometry signals obtained from these blots and used to determine S12/S30 enrichment in Figure 4 are also included and are reported

rounded to the nearest hundredths place. Source data for all panels are provided as a Source Data file.

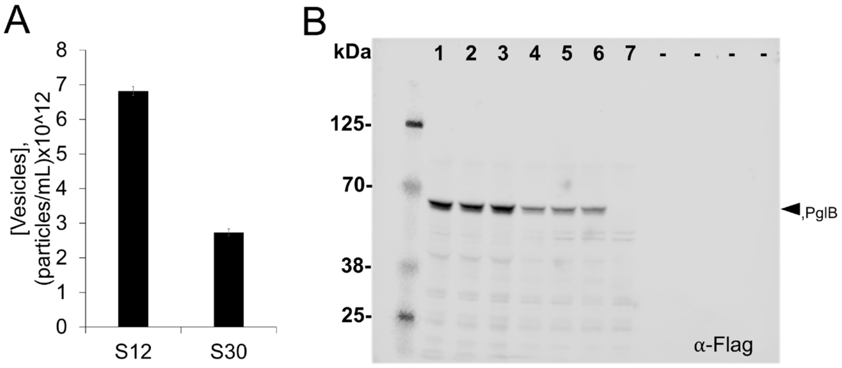

Lane	S12/S30	Extract replicate (#)	Notes
Ladder	-	-	-
1	S12	1	-
2	S12	2	-
3	S12	3	-
4	S30	1	-
5	S30	2	-
6	S30	3	-
7	S12	N/A	LLO only expression control

Supplementary Figure 7. Characterization of vesicle and PglB enrichment in CFGpS extracts. (A) Concentrations of vesicles in extracts enriched with PglB and *C. jejuni* LLO, measured via NTA. Data are presented as mean values +/- SD of n=15 replicates (3 biologically independent extracts; each examined over 5, 1-minute NTA measurements). (B) α-FLAG Western blot against PglB in an extract enriched with both PglB and LLO. Corresponding lane key is to the right of the blot and molecular weight markers are labeled in kDa to the left of the blot. The Western blot includes n=3 biologically independent S12 extracts and n=3 biologically independent S30 extracts. Source data for all panels are provided as a Source Data file.

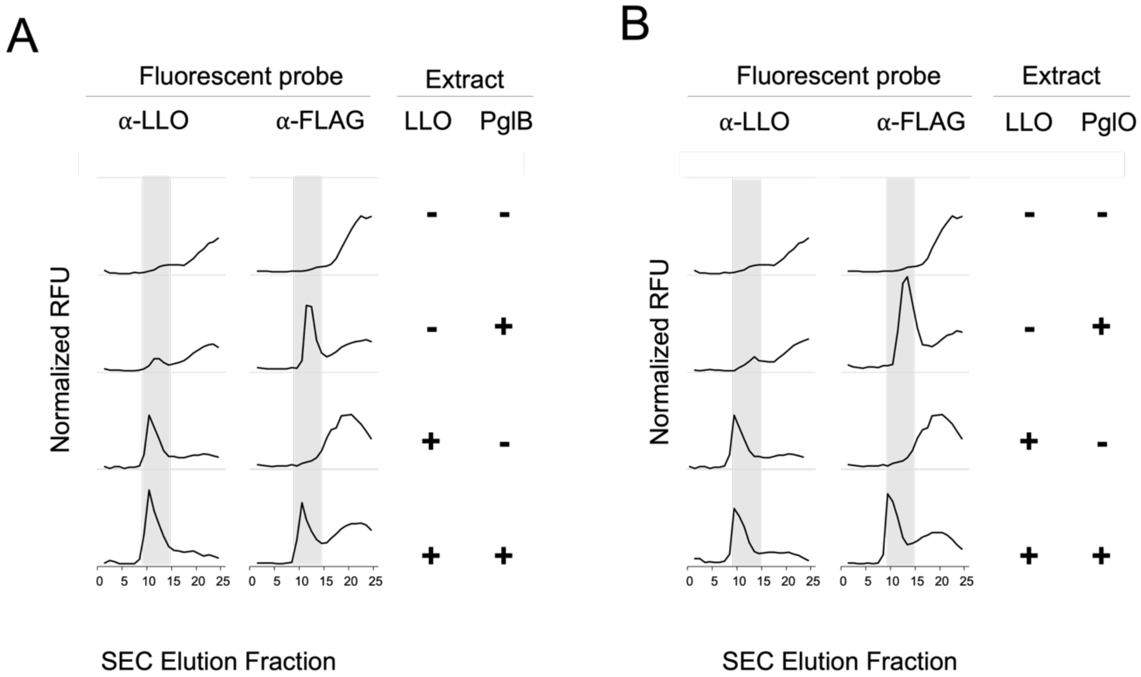

Supplementary Figure 8. Fluorescent probes confirm that glycosylation components are associated with membrane vesicles in CFE extracts. Fluorescence SEC chromatograms of vesicles probed with α-LLO and α-OST reagents are presented. Vesicle elution fraction is highlighted in gray. Analysis of extracts and controls with the *N*-linked PglB OST are presented in (A) and for the *O*-linked PglO OST in (B). A low amount of nonspecific binding of the α-LLO SBA lectin is observed and serves as a signal baseline for the LLO-containing samples. Data are representative of three independent experiments. Source data for all panels are provided as a Source Data file.

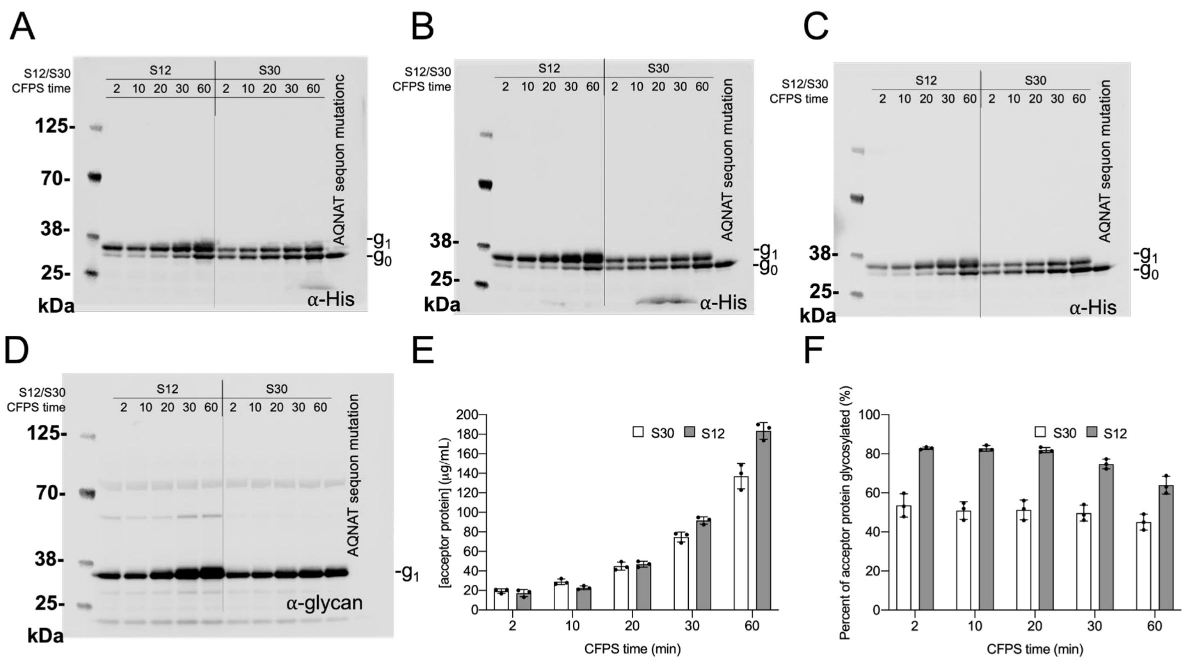

Supplementary Figure 9. Characterization of *N*-linked glycosylation in S12 and S30 CFGpS extracts. n=3 α-His Western blots against CFGpS-derived acceptor proteins are shown in (A)-(C). Each Western blot includes n=1 CFGpS reaction for each spiking condition run with a biologically independent S12 extract and a biologically independent S30 extract. This results in n=3 biologically independent reactions for each condition that were used to generate densitometry measurements. Western blots in (A)-(C) were used to calculate glycoprotein yields in Figure 5 of the main text. (D) α-glycan blot of the corresponding reactions in (A). Western blot includes CFGpS reactions run with one S12 extract and one S30 extract and is representative of 3 experiments. g_o denotes aglycosylated acceptor protein and g_1 indicates glycoprotein in (A-D) and molecular weight markers are labeled to the left of the gel. (E) Total acceptor protein produced in CFGpS reactions run with S12 (gray) and S30 (white) extracts as determined by sfGFP fluorescence converted to concentration using a ^{14}C-leucine derived standard curve, and (F) percent of acceptor protein converted to glycoprotein at each condition in CFGpS reactions run with S12 (gray) and S30 (white) extracts. Data in E-F are presented as mean values +/- SD of n=3 biologically independent CFGpS reactions. Source data for all panels are provided as a Source Data file.

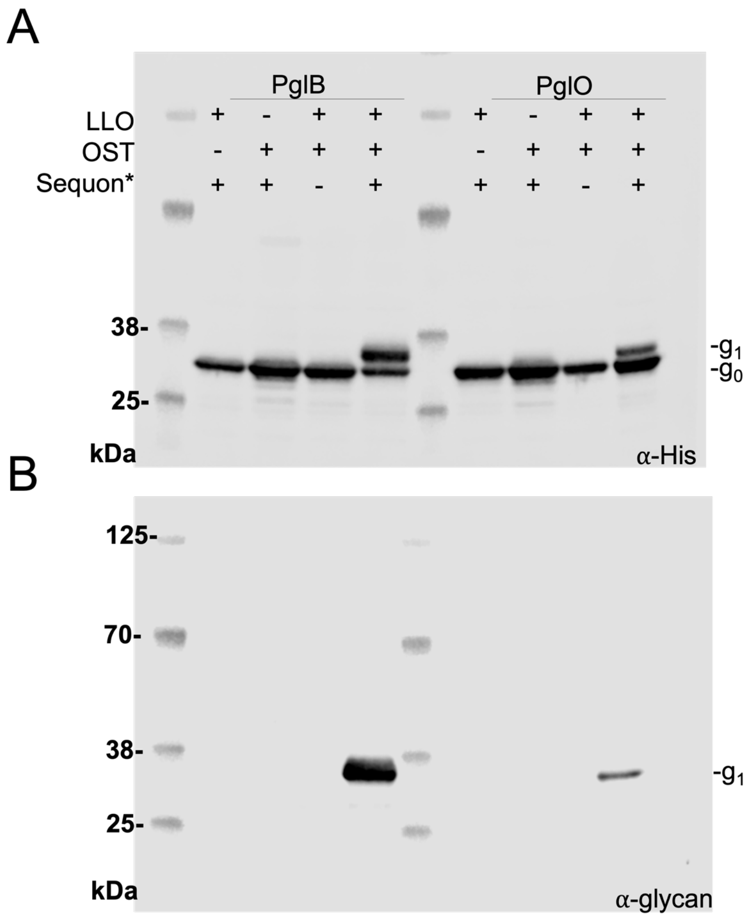

*PglB permissible sequon: DQNAT
PglB impermissible sequon: AQNAT
PglO permissible sequon: WPAAASAP
PglO impermissible sequon: WPAAAGAP

Supplementary Figure 10. Residue-specific glycosylation of acceptor proteins with permissible sequons for PglB and PglO. Generally, glycosylation preferences of PglO are less understood than those for PglB. PglB samples are used as blotting references for positive and negative controls, as the sequon specificities for positive and negative sequons are well-characterized. (A) α-His blot of CFGpS reactions run with a 5-minute CFPS time. Glycosylated band is only present when all glycosylation components and a permissible sequon are present. g_o denotes aglycosylated acceptor protein and g_1 indicates glycoprotein. Molecular weight markers are labeled. (B) α-glycan blot that corresponds to data from (A), showing a signal only for g_1 when all glycosylation components are present. Gels are representative of three independent reactions. Source data for all panels are provided as a Source Data file.

A

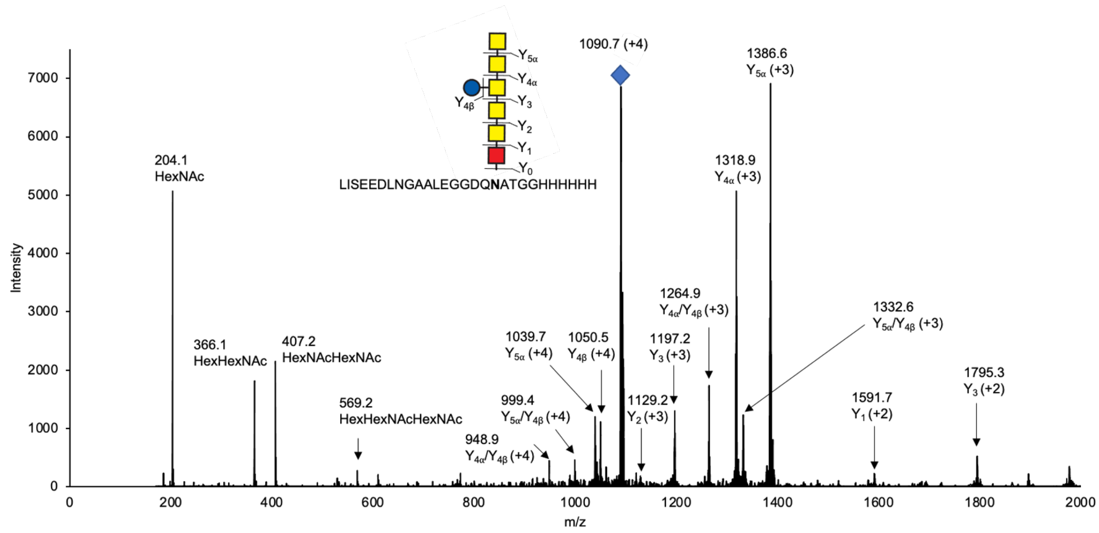

B

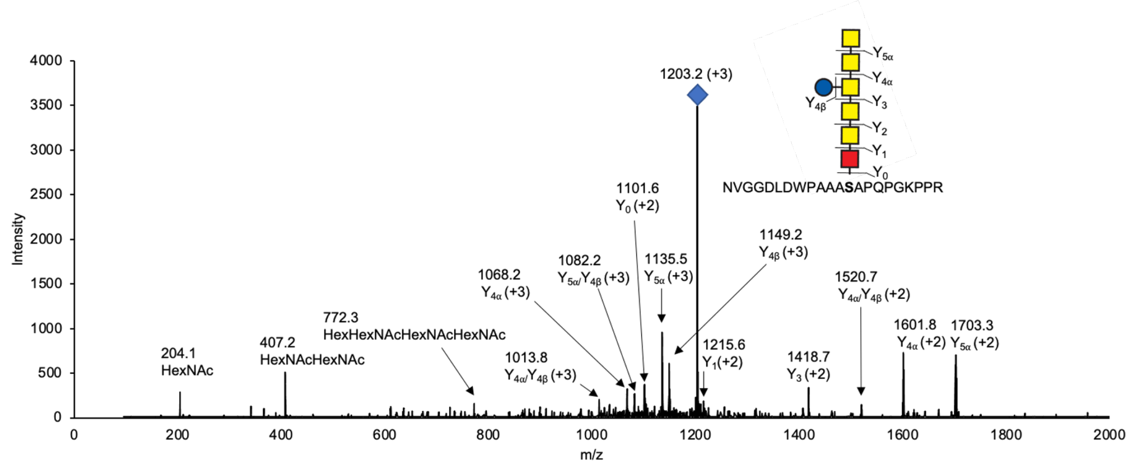

Supplementary Figure 11. LC-MS/MS of trypsin digested glycopeptides. LC-MS/MS was performed with a Bruker Elute UPLC coupled to an Impact-II UHR TOF Mass Spectrometer. (A) A quadruply-charged precursor ion (denoted with a blue diamond) was identified as the glycopeptide (LISEEDLNGAALEGGDQ**N**ATGGHHHHHH) digested from sfGFP-DQNAT (predicted *m/z* 1090.5). Glycopeptide structure is depicted in the inset. Fragmentation with an isolation window that included the entire glycopeptide isotopic envelope with 30 eV revealed glycan fragment ions as well as intact peptide with fragmented glycan characteristic of the *C. jejuni* glycan. Highest intensity peaks are labeled and are +1 charge states unless otherwise indicated. (B) A triply-charged precursor ion (denoted with a blue diamond) was identified as the glycopeptide (NVGGDLDWPAAA**S**APQPGKPPR) digested from sfGFP-MOOR (predicted *m/z* 1202.9). Glycopeptide structure is depicted in the inset. Fragmentation with an isolation window that included the entire glycopeptide isotopic envelope with 30 eV also revealed characteristic

glycan fragment ions and intact peptide with fragmented glycan characteristic of the *C. jejuni* glycan. Highest intensity peaks are labeled and are +1 charge states unless otherwise indicated. Previous reports and glycosylation site amino acid mutation studies shown in Supplementary Figure 10 strongly suggest that the glycan modification is on the bolded N and S residues within the sequons on sfGFP-DQNAT and sfGFP-MOOR glycopeptides, respectively. Source data for all panels are provided as a Source Data file.

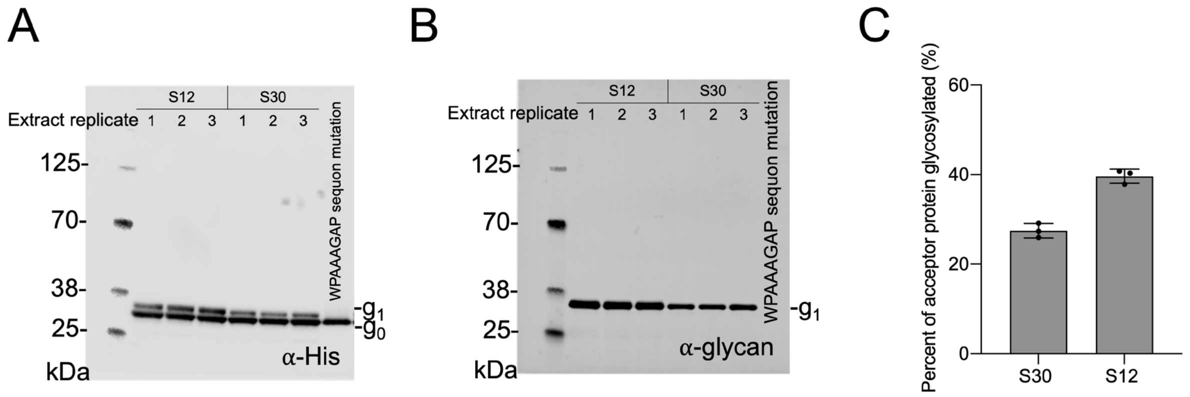

Supplementary Figure 12. Characterization of *O*-linked glycosylation in CFGpS extracts. (A) α-His Western blot of PglO CFGpS reactions run with 20-minute CFPS times and (B) α-glycan blot corresponding to data in (A). g_0 denotes aglycosylated acceptor protein and g_1 indicates glycoprotein and molecular weight markers are labeled. Each Western blot includes n=3 biologically independent CFGpS reactions for each condition that were used to generate densitometry measurements. (C) Percent of acceptor protein converted to glycoprotein. Data are presented as mean values +/- SD of n=3 biologically independent CFGpS reactions. Source data for all panels are provided as a Source Data file.

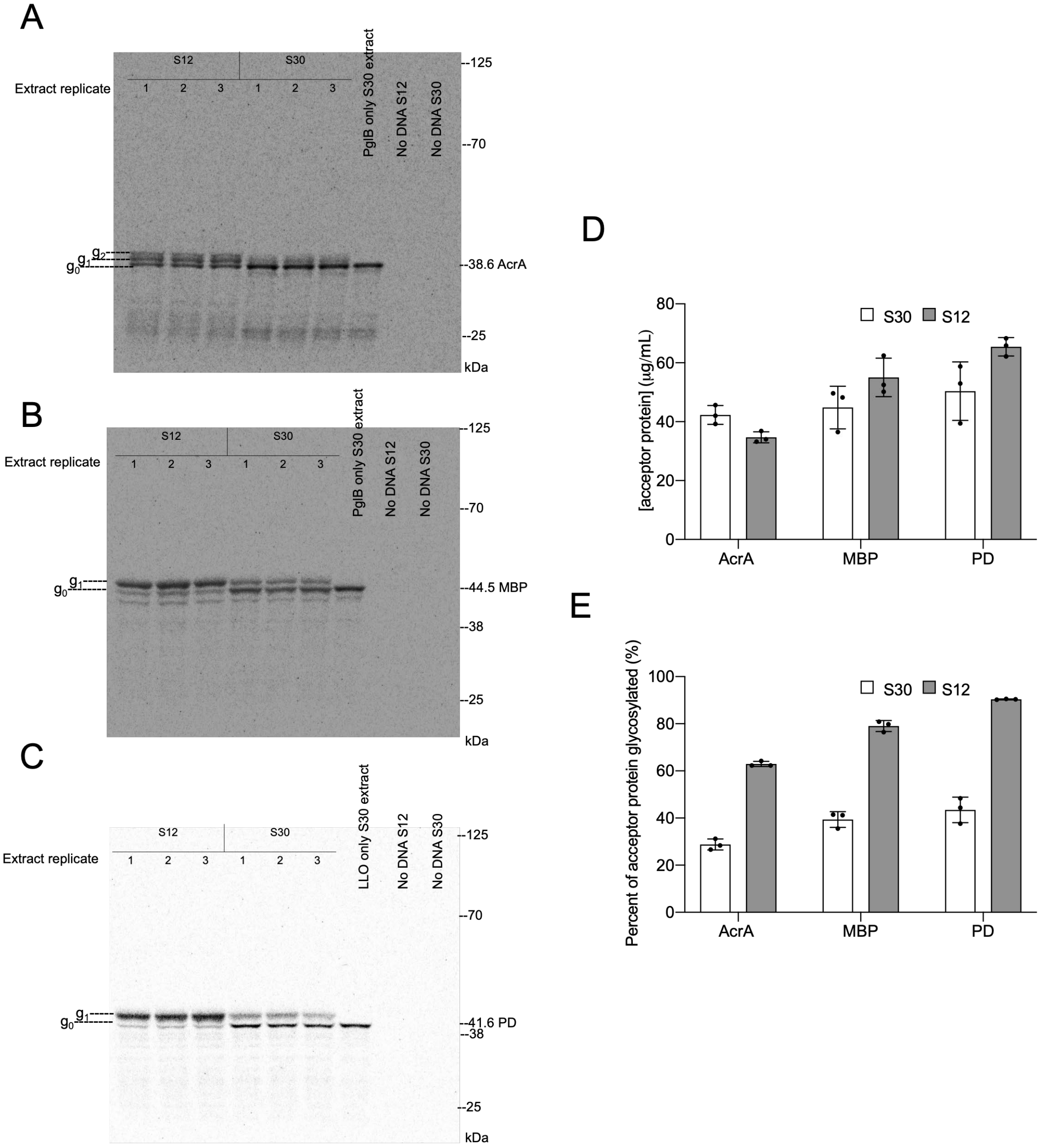

Supplementary Figure 13. Characterization of *N*-linked glycosylation with various acceptor proteins in CFGpS extracts using a ^{14}C-L incorporation assay. CFGpS reactions were run in triplicate S12 and S30 extracts with a 20 min CFPS time. (A) Autoradiogram of AcrA CFGpS reactions in n=3 biologically independent S12 and S30 extracts and negative controls. g_0 denotes aglycosylated acceptor protein, g_1 indicates singly glycosylated glycoprotein and g_2 indicates doubly glycosylated glycoprotein. (B) Autoradiogram of MBP CFGpS reactions in n=3 biologically independent S12 and S30 extracts and negative controls. g_0 denotes aglycosylated acceptor protein and g_1 indicates glycoprotein. (C) Autoradiogram of PD CFGpS reactions in n=3 biologically independent S12 and S30 extracts and negative controls. g_0 denotes aglycosylated

acceptor protein and g_1 indicates glycoprotein. Each autoradiogram includes n=3 biologically independent CFGpS reactions for each condition that were used to generate densitometry measurements. Relative molecular weights are labeled. (D) Total soluble acceptor protein produced in triplicate CFGpS reactions run with S12 (gray) and S30 (white) extracts as measured by scintillation counting of ^{14}C-labeled proteins. Data are presented as mean values +/- SD of n=3 biologically independent CFGpS reactions. (E) Percent of acceptor protein glycosylated in CFGpS reactions run with S12 (gray) and S30 (white) extracts for each acceptor protein tested as determined by densitometry of full-length product on autoradiograms. Data are presented as mean values +/- SD of n=3 biologically independent CFGpS reactions. AcrA glycoprotein was considered as the sum of singly and doubly glycosylated protein. Source data for all panels are provided as a Source Data file.

Supplementary Table 1. Information on proteins selected for extract enrichment in this study.

**Protein**	**Taxonomical origin**	**Predicted # TM helices**	**Function(s)**	**Size (kDa)**	**UniProt ID**	**UniProt hyperlink**
PglB	*Campylobacter jejuni*	13	Catalyzes *N*-linked glycosylation	82	Q5HTX9	https://www.uniprot.org/uniprot/Q5HTX9
PglO	*Neisseria gonorrhoeae*	11	Catalyzes *O*-linked glycosylation	68	Q5FA54	https://www.uniprot.org/uniprot/Q5FA54
NarX	*Escherichia coli*	2	Signal transduction for nitrate biosensing	68	P0AFA2	https://www.uniprot.org/uniprot/P0AFA2
Proteorhodopsin (PR)	Uncultured marine gamma proteobacterium EBAC3108	7	Green light absorbing proteorhodopsin	28	Q9F7P4	https://www.uniprot.org/uniprot/Q9F7P4
Cannabinoid receptor 1 (CB1)	*Homo sapiens*	7	G protein coupled receptor, molecular sensing	54	P21554	https://www.uniprot.org/uniprot/P21554
STT3D	*Leishmania major*	11	Catalyzes *N*-linked glycosylation	95	E9AET9	https://www.uniprot.org/uniprot/E9AET9

Supplementary Table 2. Acceptor protein coding sequences used in CFGpS reactions.

Sequon	Coding Sequence
pJL1-sfGFP-DQNAT	ATGAGCAAAGGTGAAGAACTGTTTACCGGCGTTGTGCCGATTCTGGTGGAACTGGATGGCGAT<br>GTGAACGGTCACAAATTCAGCGTGCGTGGTGAAGGTGAAGGCGATGCCACGATTGGCAAACT<br>GACGCTGAAATTTATCTGCACCACCGGCAAACTGCCGGTGCCGTGGCCGACGCTGGTGACCA<br>CCCTGACCTATGGCGTTCAGTGTTTTAGTCGCTATCCGGATCACATGAAACGTCACGATTTCTT<br>TAAATCTGCAATGCCGGAAGGCTATGTGCAGGAACGTACGATTAGCTTTAAAGATGATGGCAAA<br>TATAAAACGCGCGCCGTTGTGAAATTTGAAGGCGATACCCTGGTGAACCGCATTGAACTGAAA<br>GGCACGGATTTTAAAGAAGATGGCAATATCCTGGGCCATAAACTGGAATACAACTTTAATAGCC<br>ATAATGTTTATATTACGGCGGATAAACAGAAAAATGGCATCAAAGCGCAGTTTACCGTTCGCCA<br>TAACGTTGAAGATGGCAGTGTGCAGCTGGCAGATCATTATCAGCAGAATACCCCGATTGGTGA<br>TGGTCCGGTGCTGCTGCCGGATAATCATTATCTGAGCACGCAGACCGTTCTGTCTAAAGATCC<br>GAACGAAAAAGGCACGCGGGACCACATGGTTCTGCACGAATATGTGAATGCGGCAGGTATTAC<br>GCTAGGTGCGGCCGCAGAACAAAAACTCATCTCAGAAGAGGATCTGAATGGGGCCGCACTCG<br>AGGGTGGCGATCAGAACGCGACCGGCGGTCATCACCATCATCACCATTAA
pJL1-sfGFP AQNAT	ATGAGCAAAGGTGAAGAACTGTTTACCGGCGTTGTGCCGATTCTGGTGGAACTGGATGGCGAT<br>GTGAACGGTCACAAATTCAGCGTGCGTGGTGAAGGTGAAGGCGATGCCACGATTGGCAAACT<br>GACGCTGAAATTTATCTGCACCACCGGCAAACTGCCGGTGCCGTGGCCGACGCTGGTGACCA<br>CCCTGACCTATGGCGTTCAGTGTTTTAGTCGCTATCCGGATCACATGAAACGTCACGATTTCTT<br>TAAATCTGCAATGCCGGAAGGCTATGTGCAGGAACGTACGATTAGCTTTAAAGATGATGGCAAA<br>TATAAAACGCGCGCCGTTGTGAAATTTGAAGGCGATACCCTGGTGAACCGCATTGAACTGAAA<br>GGCACGGATTTTAAAGAAGATGGCAATATCCTGGGCCATAAACTGGAATACAACTTTAATAGCC<br>ATAATGTTTATATTACGGCGGATAAACAGAAAAATGGCATCAAAGCGCAGTTTACCGTTCGCCA<br>TAACGTTGAAGATGGCAGTGTGCAGCTGGCAGATCATTATCAGCAGAATACCCCGATTGGTGA<br>TGGTCCGGTGCTGCTGCCGGATAATCATTATCTGAGCACGCAGACCGTTCTGTCTAAAGATCC<br>GAACGAAAAAGGCACGCGGGACCACATGGTTCTGCACGAATATGTGAATGCGGCAGGTATTAC<br>GCTAGGTGCGGCCGCAGAACAAAAACTCATCTCAGAAGAGGATCTGAATGGGGCCGCACTCG<br>AGGGTGGCGCTCAGAACGCGACCGGCGGTCATCACCATCATCACCATTAA
pJL1-sfGFP-MOOR	ATGAGCAAAGGTGAAGAACTGTTTACCGGCGTTGTGCCGATTCTGGTGGAACTGGATGGCGAT<br>GTGAACGGTCACAAATTCAGCGTGCGTGGTGAAGGTGAAGGCGATGCCACGATTGGCAAACT<br>GACGCTGAAATTTATCTGCACCACCGGCAAACTGCCGGTGCCGTGGCCGACGCTGGTGACCA<br>CCCTGACCTATGGCGTTCAGTGTTTTAGTCGCTATCCGGATCACATGAAACGTCACGATTTCTT<br>TAAATCTGCAATGCCGGAAGGCTATGTGCAGGAACGTACGATTAGCTTTAAAGATGATGGCAAA<br>TATAAAACGCGCGCCGTTGTGAAATTTGAAGGCGATACCCTGGTGAACCGCATTGAACTGAAA<br>GGCACGGATTTTAAAGAAGATGGCAATATCCTGGGCCATAAACTGGAATACAACTTTAATAGCC<br>ATAATGTTTATATTACGGCGGATAAACAGAAAAATGGCATCAAAGCGAATTTTACCGTTCGCCAT<br>AACGTTGAAGATGGCAGTGTGCAGCTGGCAGATCATTATCAGCAGAATACCCCGATTGGTGAT<br>GGTCCGGTGCTGCTGCCGGATAATCATTATCTGAGCACGCAGACCGTTCTGTCTAAAGATCCG<br>AACGAAAAAGGCACGCGGGACCACATGGTTCTGCACGAATATGTGAATGCGGCAGGTATTACG<br>GGCTCTTCTGGAGGGTCTGGCGATCCACGCAATGTGGGTGGGGATTTGGACTGGCCGGCGG<br>CAGCGAGTGCACCTCAACCCGGTAAACCTCCTCGTCATCACCACCATCATCACTAA
pJL1-sfGFP-MOORmut	ATGAGCAAAGGTGAAGAACTGTTTACCGGCGTTGTGCCGATTCTGGTGGAACTGGATGGCGAT<br>GTGAACGGTCACAAATTCAGCGTGCGTGGTGAAGGTGAAGGCGATGCCACGATTGGCAAACT<br>GACGCTGAAATTTATCTGCACCACCGGCAAACTGCCGGTGCCGTGGCCGACGCTGGTGACCA<br>CCCTGACCTATGGCGTTCAGTGTTTTAGTCGCTATCCGGATCACATGAAACGTCACGATTTCTT<br>TAAATCTGCAATGCCGGAAGGCTATGTGCAGGAACGTACGATTAGCTTTAAAGATGATGGCAAA<br>TATAAAACGCGCGCCGTTGTGAAATTTGAAGGCGATACCCTGGTGAACCGCATTGAACTGAAA<br>GGCACGGATTTTAAAGAAGATGGCAATATCCTGGGCCATAAACTGGAATACAACTTTAATAGCC<br>ATAATGTTTATATTACGGCGGATAAACAGAAAAATGGCATCAAAGCGAATTTTACCGTTCGCCAT<br>AACGTTGAAGATGGCAGTGTGCAGCTGGCAGATCATTATCAGCAGAATACCCCGATTGGTGAT<br>GGTCCGGTGCTGCTGCCGGATAATCATTATCTGAGCACGCAGACCGTTCTGTCTAAAGATCCG<br>AACGAAAAAGGCACGCGGGACCACATGGTTCTGCACGAATATGTGAATGCGGCAGGTATTACG<br>GGCTCTTCTGGAGGGTCTGGCGATCCACGCAATGTGGGTGGGGATTTGGACTGGCCGGCGG<br>CAGCGGGTGCACCTCAACCCGGTAAACCTCCTCGTCATCACCACCATCATCACTAA

pJL1-AcrA	ATGAAAGAAGAAGCACCAAAAATACAAATGCCGCCTCAACCTGTAACAACCATGAGTGCTAAAT<br>CTGAAGATTTACCACTTAGTTTTACTTACCCTGCTAAACTTGTCAGTGATTATGATGTCATTATAA<br>AACCTCAAGTTAGCGGCGTAATAGTAAATAAACTTTTTAAAGCTGGAGATAAGGTAAAAAAAGG<br>ACAAACATTATTTATTATAGAACAAGATAAATTTAAAGCTAGTGTTGATTCAGCTTACGGACAGG<br>CTTTAATGGCTAAGGCAACTTTCGAAAATGCAAGCAAGGATTTTAATCGTTCTAAAGCTCTTTTT<br>AGCAAAAGTGCAATCTCTCAAAAAGAATACGACTCTTCTCTTGCTACATTTAACAATTCAAAAGC<br>TAGTCTAGCAAGTGCTAGAGCACAGCTTGCAAATGCAAGAATTGATCTAGATCATACCGAGATA<br>AAAGCTCCTTTTGATGGTACTATAGGAGATGCTTTAGTTAATATAGGAGATTATGTAAGTGCTTC<br>AACAACTGAACTAGTTAGAGTTACAGATTTAAATCCTATTTACGCAGATTTCTTTATTTCAGATAC<br>AGATAAACTAAATTTAGTCCGCAATACTCAAAGTGGAAAATGGGATTTAGACAGCATTCATGCA<br>AATTTAAATCTTAATGGAGAAACCGTTCAAGGCAAACTTTATTTTATTGATTCGGTTATAGATGCT<br>AATAGTGGAACAGTAAAAGCCAAAGCCGTATTTGATAACAATAACTCAACACTTTTACCGGGTG<br>CTTTTGCAACAATTACCTCAGAAGGTTTTATACAAAAAAATGGCTTTAAAGTGCCTCAAATAGGT<br>GTTAAACAAGATCAAAATGATGTTTATGTTCTTCTCGTTAAAAATGGAAAAGTAGAAAAATCTTCT<br>GTACATATAAGCTACCAAAACAATGAATACGCCATTATTGACAAAGGATTGCAAAATGGCGATA<br>AAATCATTTTAGATAACTCTAAAAAAATTCAAGTTGGTAGCGAAGTTAAAGAAATTGGAGCACAA<br>CACCATCACCATCACCATTAA
pJL1-<br>MBP-<br>DQNAT	ATGAAAATCGAAGAAGGTAAACTGGTAATCTGGATTAACGGCGATAAAGGCTATAACGGTCTCG<br>CTGAAGTCGGTAAGAAATTCGAGAAAGATACCGGAATTAAAGTCACCGTTGAGCATCCGGATA<br>AACTGGAAGAGAAATTCCCACAGGTTGCGGCAACTGGCGATGGCCCTGACATTATCTTCTGGG<br>CACACGACCGCTTTGGTGGCTACGCTCAATCTGGCCTGTTGGCTGAAATCACCCCGGACAAAG<br>CGTTCCAGGACAAGCTGTATCCGTTTACCTGGGATGCCGTACGTTACAACGGCAAGCTGATTG<br>CTTACCCGATCGCTGTTGAAGCGTTATCGCTGATTTATAACAAAGATCTGCTGCCGAACCCGCC<br>AAAAACCTGGGAAGAGATCCCGGCGCTGGATAAAGAACTGAAAGCGAAAGGTAAGAGCGCGC<br>TGATGTTCAACCTGCAAGAACCGTACTTCACCTGGCCGCTGATTGCTGCTGACGGGGGTTATG<br>CGTTCAAGTATGAAAACGGCAAGTACGACATTAAAGACGTGGGCGTGGATAACGCTGGCGCGA<br>AAGCGGGTCTGACCTTCCTGGTTGACCTGATTAAAAACAAACACATGAATGCAGACACCGATTA<br>CTCCATCGCAGAAGCTGCCTTTAATAAAGGCGAAACAGCGATGACCATCAACGGCCCGTGGGC<br>ATGGTCCAACATCGACACCAGCAAAGTGAATTATGGTGTAACGGTACTGCCGACCTTCAAGGG<br>TCAACCATCCAAACCGTTCGTTGGCGTGCTGAGCGCAGGTATTAACGCCGCCAGTCCGAACAA<br>AGAGCTGGCGAAAGAGTTCCTCGAAAACTATCTGCTGACTGATGAAGGTCTGGAAGCGGTTAA<br>TAAAGACAAACCGCTGGGTACCGTAGCGCTGAAGTCTTACGAGGAAGAGTTGGCGAAAGATCC<br>ACGTATTGCCGCCACCATGGAAAACGCCCAGAAAGGTGAAATCATGCCGAACATCCCGCAGAT<br>GTCCGCTTTCTGGTATGCCGTGCGTACTGCGGTGATCAACGCCGCCAGCGGTCGTCAGACTG<br>TCGATGAAGCCCTGAAAGACGCGCAGACTCGTATCACCAAGCTAGGTGCGGCCGCAGAACAA<br>AAACTCATCTCAGAAGAGGATCTGAATGGGGCCGCACTCGAGGGTGGCGATCAGAACGCGAC<br>CGGCGGTCATCACCATCATCACCATTAA
pJL1-PD-<br>DQNAT	ATGAAGTCAGACAAGATCATTATCGCGCATCGCGGTGCCTCCGGCTACTTGCCGGAGCATACT<br>TTGGAGAGCAAAGCCTTAGCATTTGCCCAACAAGCGGATTACTTGGAACAAGACTTGGCCATG<br>ACTAAGGATGGGCGTCTGGTGGTGATTCACGACCATTTTCTTGATGGTCTTACGGATGTAGCTA<br>AGAAGTTTCCGCATCGCCATCGTAAGGACGGGCGCTATTATGTAATCGATTTTACCTTGAAGGA<br>AATTCAAAGCTTGGAGATGACCGAAAACTTTGAGACGAAGGATGGGAAGCAAGCGCAAGTCTA<br>CCCGAATCGTTTTCCCTTATGGAAAAGCCATTTTCGCATCCATACTTTTGAGGATGAGATTGAG<br>TTTATCCAAGGGCTGGAAAAGTCTACTGGCAAGAAAGTTGGGATCTATCCTGAGATCAAGGCA<br>CCGTGGTTTCATCATCAAAACGGTAAGGACATCGCCGCTGAGACCTTAAAAGTACTGAAGAAG<br>TATGGCTACGATAAAAAGACTGACATGGTTTACCTTCAAACGTTCGATTTTAATGAGTTGAAACG<br>TATTAAAACAGAATTATTGCCCCAGATGGGGATGGATTTGAAGTTGGTTCAGTTAATCGCCTAC<br>ACCGACTGGAAGGAAACGCAGGAGAAGGATCCCAAAGGATACTGGGTGAATTATAATTATGAT<br>TGGATGTTCAAGCCAGGAGCAATGGCTGAAGTAGTAAAATATGCGGATGGTGTAGGCCCCGG<br>CTGGTATATGCTTGTGAACAAAGAAGAAAGCAAGCCTGATAATATCGTCTATACTCCCTTAGTC<br>AAAGAACTGGCCCAATATAACGTAGAAGTTCACCCTTACACCGTCCGTAAGGACGCCTTACCG<br>GAGTTTTTTACTGACGTCAATCAAATGTATGATGCCTTGCTGAACAAGAGTGGAGCGACAGGC<br>GTCTTCACCGACTTTCCAGACACTCTAGGTGCGGCCGCAGAACAAAAACTCATCTCAGAAGAG<br>GATCTGAATGGGGCCGCACTCGAGGGTGGCGATCAGAACGCGACCGGCGGTCATCACCATCA<br>TCACCATTAA

Supplementary Table 3. Strains and plasmids used in this study.

Strain/Plasmid	Description	Antibiotic Resistance	Reference
E. coli CLM24	W3110, ΔWaaL	N/A	(1)
pJL1-sfGFP	pJL1 plasmid encoding superfolder GFP	Kan50	(2)
pSF-CjPglB	pSN18 derivative encoding *C. jejuni* PglB with C-terminal FLAG epitope tag	Carb100	(3)
pSF-NgPglO	pSN18 derivative encoding *N. gonorrhoeae* PglO with C-terminal FLAG epitope tag	Carb100	This study
pSF-EcNarX	pSN18 derivative encoding *E. coli* NarX with C-terminal FLAG epitope tag	Carb100	This study
pSF-PR	pSN18 derivative encoding Uncultured marine gamma proteobacterium EBAC3108 proteorhodopsin with C-terminal FLAG epitope tag	Carb100	This study
pSF-HsCB1	pSN18 derivative encoding *H. sapiens* Cannabinoid receptor 1 with C-terminal FLAG epitope tag	Carb100	This study
pSF-LmSTT3D	pSN18 derivative encoding *L. major* STT3D with C-terminal FLAG epitope tag	Carb100	This study
pSF-sfGFP	pSN18 derivative encoding superfolder GFP with C-terminal FLAG epitope tag	Carb100	This study
pMW07-pglΔB	pMW07 plasmid encoding *C. jejuni* protein glycosylation locus (pgl) with complete in-frame deletion of CjPglB	Cm34	(3)
pJL1-sfGFP-DQNAT	pJL1 plasmid encoding superfolder GFP modified at the C-terminus with 30 amino acids containing an optimal DQNAT sequon followed by a 6x-His tag	Kan50	This study, see **Table S2** for sequence
pJL1-sfGFP-AQNAT	pJL1 plasmid encoding superfolder GFP modified at the C-terminus with 30 amino acids containing a non-permissible AQNAT sequon followed by a 6x-His tag	Kan50	This study, see **Table S2** for sequence
pJL1-sfGFP-MOOR	pJL1 plasmid encoding superfolder GFP modified at the C-terminus with 32 amino acids containing the minimum optimal *O*-linked recognition site (MOOR)[4] followed by a 6x-His tag	Kan50	This study, see **Table S2** for sequence
pJL1-sfGFP-MOORmut	pJL1 plasmid encoding superfolder GFP modified at the C-terminus with 32 amino acids containing a non-permissible minimum optimal *O*-linked recognition site (MOORmut) followed by a 6x-His tag	Kan50	This study, see **Table S2** for sequence
pJL1-AcrA	pJL1 plasmid encoding wt AcrA from *C. jejuni c*ontaining 2 native internal glycosylation sites recognized by PglB and a C-terminal 6x-His tag	Kan50	(5)
pJL1-MBP-DQNAT	pJL1 plasmid encoding *E. coli* maltose binding protein (MBP) modified at the C-terminus with 30 amino acids containing an optimal DQNAT sequon followed by a 6x-His tag	Kan 50	This study, see **Table S2** for sequence. Note coding sequence of acceptor protein is used in a previous study with a different sequon (6).
pJL1-PD-DQNAT	pJL1 plasmid encoding *H. influenzae p*rotein D (PD) modified at the C-terminus with 30 amino acids containing an optimal DQNAT sequon followed by a 6x-His tag	Kan50	This study, see **Table S2** for sequence. Note coding sequence of acceptor protein is used in a previous study with a different sequon (6).

Supplementary Table 4. Information on primers used to construct plasmids made for this study.

Primer	Sequence	Description
1	GGAGGAATTAACCATGGCATCTAGTATGAGCGCAGAAACCACC	Forward primer to amplify NgPglO-Flag with homology to pSF backbone at the N-terminus.
2	CGTCCTTGTAGTCTGCTCCTGCAGGTTTACACGGTTTGGTTTCCGG	Reverse primer to amplify NgPglO-Flag with homology to pSF backbone at the N-terminus.
3	CCTGCAGGAGCAGACTAC	Forward primer to amplify pSF backbone with Flag tag.
4	ACTAGATGCCATGGTTAATTCCTCC	Reverse primer to amplify pSF backbone with Flag tag.
5	CCCTCTAGAAATAATTTTGTTTAACTTTAAGAAGGAGATATACATATG	Forward primer to amplify sfGFP with homology to the pJL1 backbone and linker (used between MOOR/MOORmut and sfGFP).
6	GCCAGACCCTCCAGAAGAGCCCGTAATACCTGCCGCATTCACATATTC	Reverse primer to amplify sfGFP with homology to the pJL1 backbone and linker (used between MOOR/MOORmut and sfGFP).
7	GGCTCTTCTGGAGGGTCTGGCGATCCACGCAATGTGGGTGG	Forward primer to amplify pJL1 with MOOR/MOORmut tag and linker used between tag and sfGFP.
8	ATGTATATCTCCTTCTTAAAGTTAAACAAAATTATTTCTAGAGGG	Reverse primer to amplify pJL1 with MOOR/MOORmut tag and linker used between tag and sfGFP.

Supplementary Methods

Zeta Potential Analysis

Zeta potential measurements were performed in triplicate (n=3) for 15 scans per measurement on a Zetasizer Nano ZS (Malvern Instruments Ltd.) using standard settings at room temperature and in disposable zeta potential cuvettes (Malvern Instruments Ltd., UK DTS1070).

Supplementary References

1. Feldman, M. F. et al. Engineering N-linked protein glycosylation with diverse O antigen lipopolysaccharide structures in Escherichia coli. Proc. Natl. Acad. Sci. U. S. A. (2005). doi:10.1073/pnas.0500044102
2. Stark, J. C. et al. BioBitsTM Bright: A fluorescent synthetic biology education kit. Sci. Adv. 4, (2018).
3. Ollis, A. A., Zhang, S., Fisher, A. C. & DeLisa, M. P. Engineered oligosaccharyltransferases with greatly relaxed acceptor-site specificity. Nat. Chem. Biol. 10, 816–822 (2014).
4. Pan, C. et al. Biosynthesis of conjugate vaccines using an O-linked glycosylation system. MBio 7, (2016).
5. Schoborg, J. A. et al. A cell-free platform for rapid synthesis and testing of active oligosaccharyltransferases. Biotechnology and Bioengineering (2017). doi:10.1002/bit.26502
6. Stark, J. C. et al. On-demand, cell-free biomanufacturing of conjugate vaccines at the point-of-care. Sci. Adv. 7, eabe9444 (2021).
