# Supplementary material for: Improving cell-free glycoprotein synthesis by characterizing and enriching native membrane vesicles
Source: Nat Commun. 2021 Apr 22;12:2363. doi: 10.1038/s41467-021-22329-3 (PMC8062659; doi:10.1038/s41467-021-22329-3)
Supplement: Supplementary file 2 — Reporting Summary [file 41467_2021_22329_MOESM2_ESM.pdf]

nature research

Corresponding author(s): Michael C Jewett

Last updated by author(s): Dec 21, 2020

Reporting Summary

Nature Research wishes to improve the reproducibility of the work that we publish. This form provides structure for consistency and transparency in reporting. For further information on Nature Research policies, see Authors & Referees and the Editorial Policy Checklist .

Statistics

For all statistical analyses, confirm that the following items are present in the figure legend, table legend, main text, or Methods section.

n/a	Confirmed	
☐	☒	The exact sample size (*n*) for each experimental group/condition, given as a discrete number and unit of measurement
☐	☒	A statement on whether measurements were taken from distinct samples or whether the same sample was measured repeatedly
☒	☐	The statistical test(s) used AND whether they are one- or two-sided<br>*Only common tests should be described solely by name; describe more complex techniques in the Methods section.*
☒	☐	A description of all covariates tested
☐	☒	A description of any assumptions or corrections, such as tests of normality and adjustment for multiple comparisons
☐	☒	A full description of the statistical parameters including central tendency (e.g. means) or other basic estimates (e.g. regression coefficient) AND variation (e.g. standard deviation) or associated estimates of uncertainty (e.g. confidence intervals)
☒	☐	For null hypothesis testing, the test statistic (e.g. *F*, *t*, *r*) with confidence intervals, effect sizes, degrees of freedom and *P* value noted<br>*Give P values as exact values whenever suitable.*
☒	☐	For Bayesian analysis, information on the choice of priors and Markov chain Monte Carlo settings
☒	☐	For hierarchical and complex designs, identification of the appropriate level for tests and full reporting of outcomes
☒	☐	Estimates of effect sizes (e.g. Cohen's *d*, Pearson's *r*), indicating how they were calculated

Our web collection on statistics for biologists contains articles on many of the points above.

Software and code

Policy information about availability of computer code

Data collection	All data were collected using stated instruments and associated commercially available software. No in-house algorithms or softwares were used to collect the data. Commercial software used includes: Image Studio v. 5.2.5 (LI-COR Biosciences) for obtaining Western Blot images, Zetasizer Software V7.13 (Malvern Panalytical) & NanoSight NTA 3.4 Build 3.4.003 (Malvern Panalytical) for vesicle analysis, Gen5 v. 2.09 (BioTek) for fluorescence measurements, Gatan Digital Micrograph version 1.82.305 for collecting microscopy images, Microbeta2 v. 1.0 SP1 (PerkinElmer) for collecting scintillation counting data, Typhoon FLA 7000 Control Software Version 1.2 Build 1.2.1.93 (GE Healthcare) for obtaining autoradiogram images, and Bruker Compass Hystar v. 5.0 (Bruker Daltonics, Inc.) for acquiring LC-MS/MS data .
Data analysis	All data were analyzed using commercially-available softwares. No in-house algorithms or softwares were used to analyze the data. Software for data analysis includes: Excel (Microsoft), Prism v.9.0.0 (GraphPad), Image-J v. 2.1.0/1.53 Build 5f23140693 (NIH) for analyzing autoradiograms, Image Studio Lite v. 5.2.5 (LI-COR Biosciences) for analyzing Western blots, Photoshop CC 2017.0.0, 20161012.r.53 x64 to crop and add the scale bars to electron micrographs, and Bruker Compass Data Analysis v. 4.4 (Bruker Daltonics, Inc.) for analyzing MS/MS data.

For manuscripts utilizing custom algorithms or software that are central to the research but not yet described in published literature, software must be made available to editors/reviewers. We strongly encourage code deposition in a community repository (e.g. GitHub). See the Nature Research guidelines for submitting code & software for further information.

Data

Policy information about availability of data

All manuscripts must include a data availability statement. This statement should provide the following information, where applicable:

- Accession codes, unique identifiers, or web links for publicly available datasets
- A list of figures that have associated raw data
- A description of any restrictions on data availability

All data generated or analyzed during this study are included in the manuscript or supplementary information or are available from the corresponding author upon request. Accession codes are listed for applicable genes in the supplementary information. We report no restrictions on data availability. Uniprot accession codes used in this study are: Q5HTX9 (https://www.uniprot.org/uniprot/Q5HTX9); (Q5FA54 https://www.uniprot.org/uniprot/Q5FA54); P0AFA2 (https://www.uniprot.org/uniprot/P0AFA2); Q9F7P4 (https://www.uniprot.org/uniprot/Q9F7P4); P21554 (https://www.uniprot.org/uniprot/P21554); E9AET9 (https://www.uniprot.org/uniprot/E9AET9)

Field-specific reporting

Please select the one below that is the best fit for your research. If you are not sure, read the appropriate sections before making your selection.

- [x] Life sciences
- [ ] Behavioural & social sciences
- [ ] Ecological, evolutionary & environmental sciences

For a reference copy of the document with all sections, see nature.com/documents/nr-reporting-summary-flat.pdf

Life sciences study design

All studies must disclose on these points even when the disclosure is negative.

Sample size	For quantification of vesicle sizes and concentrations, n=3 uniquely prepared extracts for each condition were analyzed. Quantification of membrane protein enrichment was performed on n=3 uniquely prepared extracts for each condition tested. Quantification of acceptor protein production and glycosylation activity from n=3 unique CFGpS reactions- wherein the extracts were prepared uniquely in triplicate- were used to enable calculation of mean and standard deviation as is regular practice in cell-free literature. Sample sizes were determined based on precedent in previous literature and not by calculations.
Data exclusions	None to report.
Replication	All attempts at replication were successful and biological triplicates of each sample were used to verify reproducibility.
Randomization	Samples were analyzed equally, then characterized fully and reported in completion. There was no requirement for randomization.
Blinding	Blinding was not relevant, as animal or human participants were not used in this study. The experiments performed in this study would not be impacted by blinding.

Reporting for specific materials, systems and methods

We require information from authors about some types of materials, experimental systems and methods used in many studies. Here, indicate whether each material, system or method listed is relevant to your study. If you are not sure if a list item applies to your research, read the appropriate section before selecting a response.

Materials & experimental systems

n/a	Involved in the study	
[ ]	[x]	Antibodies
[x]	[ ]	Eukaryotic cell lines
[x]	[ ]	Palaeontology
[x]	[ ]	Animals and other organisms
[x]	[ ]	Human research participants
[x]	[ ]	Clinical data

Methods

n/a	Involved in the study	
[x]	[ ]	ChIP-seq
[x]	[ ]	Flow cytometry
[x]	[ ]	MRI-based neuroimaging

Antibodies

Antibodies used	Western blotting and immunostaining were performed according to standard protocols using the following antibodies: 1)anti-6x His tag-antibody (Abcam, ab1187), 2) anti-DDDDK tag (FLAG tag) antibody (Abcam 2493), 3) goat anti-Rabbit IgG IRDye 680RD (LI-COR, 925-68071), 4) SBA-AlexaFluor 594 (Invitrogen, 32462), 5) anti-FLAG-DyLight 488 antibody (Invitrogen, MA191878D488) and 6) C. jejuni heptasaccharide glycan-specific antiserum hR6 (a gift from Dr. Markus Aebi, ETH-Zurich).
Validation	Antibodies listed 1-5 are comprehensively validated for quality and performance by the vendors, and quality control information

for each antibody batch is provided by the vendors upon reagent arrival. Antibodies 1 and 2 were used as primary antibodies for Western blotting and are tested for Western blotting application by Abcam (manufacturer). Antibody 3 was used as a secondary antibody for Western blotting and has been "specifically tested and qualified for Western blot and In-Cell Western™ assay applications" by Licor. Antibody 6, the hR6 serum was used as a primary antibody for Western blotting, which has been rigorously validated by the providing lab (Dr. Markus Aebi, ETH Zurich) and has been used in many publications. We have previously published multiple manuscripts that make use of hR6. All antibodies used for Western blotting were validated for binding at chosen dilutions and ensured to be in the range of detection of the Licor instrument. Antibodies 4 and 5 were used for immunostaining vesicles before SEC purification and were chosen to have orthogonal excitation and emission wavelengths. The concentrations of fluorescently-conjugated antibodies added to lysate were validated against negative and positive lysate controls for optimum binding and signal. For Antibody 4: "Lectin soybean agglutinin (SBA) selectively binds terminal α- and β-N-acetylgalactosamine and galactopyranosyl residues" and the fluorophore has an excitation/emission of 590/617. For Antibody 5: "MA1-91878-D488 has been successfully used for immunofluorescence. DyLight 488 has an excitation/emission of 493/518 nm".
